# Long-term viability and function of transplanted islets macroencapsulated at high density are achieved by enhanced oxygen supply

**DOI:** 10.1038/s41598-018-23862-w

**Published:** 2018-04-25

**Authors:** Yoav Evron, Clark K. Colton, Barbara Ludwig, Gordon C. Weir, Baruch Zimermann, Shiri Maimon, Tova Neufeld, Nurit Shalev, Tali Goldman, Assaf Leon, Karina Yavriyants, Noa Shabtay, Tania Rozenshtein, Dimitri Azarov, Amanda R. DiIenno, Anja Steffen, Paul de Vos, Stefan R. Bornstein, Uriel Barkai, Avi Rotem

**Affiliations:** 1 Beta-O2 Technologies, Ltd., 11 Amal Street, P.O. Box 11793, Rosh-Ha’ain, 4809900 Israel; 2 Department of Chemical Engineering, Massachusetts Institute of Technology, 77 Massachusetts Avenue, Cambridge, MA 02139 USA; 3 University Hospital Carl Gustav Carus, Department of Medicine III, 74 Fetscher Street, Dresden, D-01307 Germany; 4 Section of Islet Transplantation and Cell Biology, Joslin Diabetes Center, Research Division, One Joslin Place, Boston, MA 02215 USA; 5 Department of Pathology and Laboratory Medicine, Section of Immunoendocrinology, University Medical Center Groningen, Hanzeplein 1, 9700 RB Groningen, The Netherlands

## Abstract

Transplantation of encapsulated islets can cure diabetes without immunosuppression, but oxygen supply limitations can cause failure. We investigated a retrievable macroencapsulation device wherein islets are encapsulated in a planar alginate slab and supplied with exogenous oxygen from a replenishable gas chamber. Translation to clinically-useful devices entails reduction of device size by increasing islet surface density, which requires increased gas chamber pO_2._ Here we show that islet surface density can be substantially increased safely by increasing gas chamber pO_2_ to a supraphysiological level that maintains all islets viable and functional. These levels were determined from measurements of pO_2_ profiles in islet-alginate slabs. Encapsulated islets implanted with surface density as high as 4,800 islet equivalents/cm^3^ in diabetic rats maintained normoglycemia for more than 7 months and provided near-normal intravenous glucose tolerance tests. Nearly 90% of the original viable tissue was recovered after device explantation. Damaged islets failed after progressively shorter times. The required values of gas chamber pO_2_ were predictable from a mathematical model of oxygen consumption and diffusion in the device. These results demonstrate feasibility of developing retrievable macroencapsulated devices small enough for clinical use and provide a firm basis for design of devices for testing in large animals and humans.

## Introduction

Islet transplantation can functionally cure Type 1 diabetes but requires immunosuppression. Islet encapsulation has promise for eliminating immunosuppression, but improvements in performance are needed^1^. Foremost amongst the outstanding problems is oxygen supply limitation^2^, which is a primary cause of failure. Donor islets are isolated from pancreatic tissue by enzymatic and mechanical processing, which disrupts their blood supply. Transplanted islets rely solely on diffusion for oxygen and nutrient supply, secretion of hormones, and removal of waste products. Oxygen is the first species to become diffusion limited^3^. Oxygen limitations occur in naked islets and in islets encapsulated in capsules or in retrievable macroencapsulation devices of various configurations and have been investigated with theoretical models and observed by experimental measurements^2^. Protection of islets from the host immune system by encapsulation aggravates the problem and further impairs islet viability and function. In addition, hypoxia followed by cell necrosis intensifies the immune response to transplanted tissue^4^.

Various approaches have been studied to enhance oxygen supply to encapsulated islets and other therapeutic cells: (1) reduction of diffusion distances in the device and islet tissue; (2) increase of the oxygen permeability of encapsulating material; (3) induction of neovascularization adjacent to the immunobarrier device with angiogenic proteins or vascularizing membranes to bring blood flow close to the tissue; and (4) provision of exogenous oxygen to produce a direct increase in oxygen concentration adjacent to the encapsulated tissue (2). Previous methods for providing exogenous oxygen include continuous local oxygen generation by *in situ* water electrolysis^5,6^ or photosynthesis^7,8^ or transient generation by hydrolytically-activated decomposition of solid peroxides^9^. We have used a simpler approach with a macroencapsulation device designed to enhance the supply of oxygen to the implanted islets while protecting the islets from immune attack. The device contained islets immobilized within a flat alginate slab overlain by immunobarriers that prevent or minimize the cellular and humoral immune response to donor tissue. The slab was supplied with oxygen by diffusion through an oxygen-permeable membrane from a gas mixture in an adjacent chamber replenished daily through an externalized port. In previous work, devices containing either isogeneic or allogeneic islets immobilized at relatively low surface density (as low as about 1,400 IEQ/cm^2^ with air in the gas chamber) achieved normoglycemia in diabetic rats^10,11^, as did xenogeneic islets in minipigs^12^. A very low, sub-clinical dose of allogeneic human islets produced persistent graft function in a human with regulated insulin secretion and preservation of islet morphology without immunosuppression^13^.

To translate these findings to regular clinical application, we aimed to minimize device size. Because the en face surface area required is inversely proportional to islet surface density (IEQ/cm^2^) that can be supported, our goal was to increase density by increasing oxygen partial pressure (pO_2_) in the internal gas chamber. We hypothesized that higher islet surface density can be achieved without damage from exposure to high pO2^6^ by using the minimum chamber pO_2_ required to support islets furthest away from the chamber. As O_2_ diffuses radially inward and is consumed by cells in an islet, its concentration decreases. For a spherical human islet equivalent (IEQ) containing 1,560 cells^14^ and a 150-μm diameter, the outer islet surface should be at an oxygen partial pressure (pO_2_) of about 45–50 mmHg to maintain full functionality of all beta cells throughout the islet^2,15–17^.

Here we show that a density of 4,800 IEQ/cm^2^ for islets within the alginate slab of the device can be achieved by increasing oxygen concentration in the gas chamber to p0_2_ $\bar{ > }$ 300 mmHg, thereby enabling the use of a much smaller device for implantation. pO_2_ profiles measured in the slab *in vitro* were used to determine the minimum gas chamber pO_2_ that maintains the local bulk pO_2_ ≥ 50 mmHg throughout the slab at all times. These data were consistent with prediction of gas chamber pO_2_ from a simplified mathematical model describing oxygen diffusion and consumption in the slab. The required conditions were used in isogeneic implantations in diabetic rats with densities ranging up to about 4,800 IEQ/cm^2^ at constant islet loading of about 2,400 + 200 IEQ. Animals remained normoglycemic after implant periods ranging up to 229 days before elective termination. Intravenous glucose tolerance tests were comparable to those of nondiabetic animals, and oxygen consumption rate (OCR) measurements with islet slabs revealed that explanted islets retained an average of 88% of their pre-implantation value, thereby demonstrating that the transplanted islets retained their viability and function over long periods of implantation. Lastly, duration of normoglycemia correlated with generalized islet quality assessment parameters in a manner similar to that obtained with naked rat islet transplants in mice. These data demonstrate that little islet damage was incurred by use of elevated pO_2_ in the gas chamber over the range studied, and the results provide a firm basis for the design and application of devices in future large animal and clinical studies.

## Results

**Figure 1:**
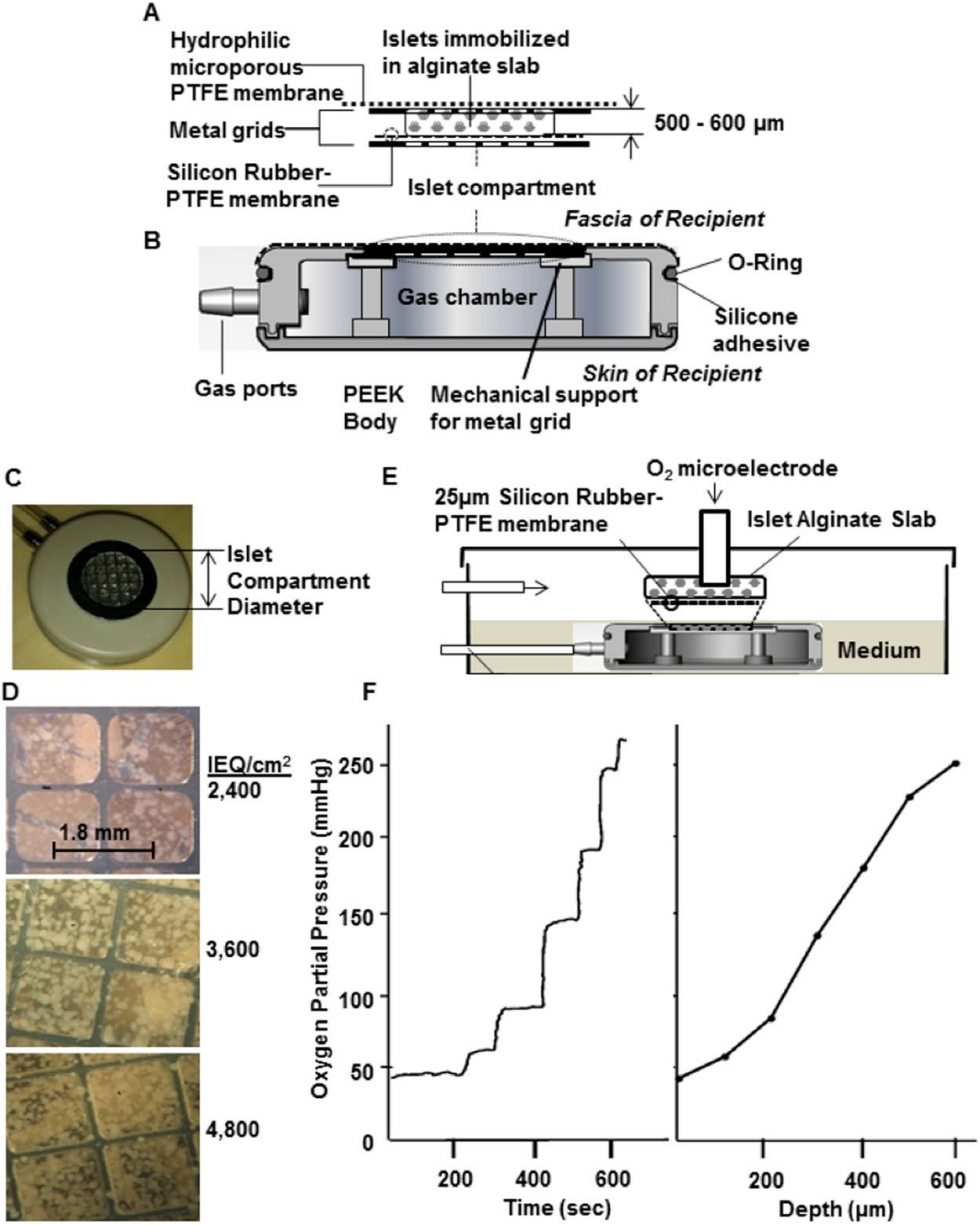
βAir device and system to measure oxygen profile within slabs. (**A**) Islet compartment with islets embedded in a 500–600 μm thick alginate slab reinforced on each side with a stainless steel grid, outer face overlain with alginate-filled PTFE membrane, which interfaces with host tissue, interior face covered by oxygen-permeable silicone rubber-PTFE membrane. (**B**) PEEK housing contained gas chamber and two ports connected to subcutaneously-implanted access ports. (**C**) Top view of βAir device with about 2,400 IEQ. Islet density was varied by varying diameter of islet chamber. (**D**) Top surface of devices with different islet densities, islets visible beneath top metal grid. Volume fraction of slab occupied by islets with about 2,400, 3,600, and 4,800 IEQ/cm^2^ was 8.5, 12.7, and 17.0%, respectively, at slab thickness of 500 μm. (**E**) Oxygen profiles in islet compartment measured with modified βAir device (lacking alginate-filled membrane and top metal grid) placed in chamber, 37 °C, pO_2_ 40 mmHg to simulate microvasculature). Device was immersed with very thin layer of medium over slab, gas chamber purged with mixtures at various pO_2_ (outlet port not shown). pO_2_ microelectrode under control of micromanipulator inserted into slab, traversed in stepwise increments. (**F**) Typical oxygen profile within slab. About 2,400 IEQ with OCR of 3.5 pmol/IEQ/min immobilized in a 600-μm alginate slab at a density of 4,800 IEQ/cm^2^. pO_2_ electrode was inserted at immobilized islet slab-medium interface and moved sequentially at increments of 100 μm down to the gas permeable membrane. Left Panel: Raw data. Right Panel: pO_2_ profile calculated from steady-state data in left panel.

One device design was used in all experiments reported here. Islets were immobilized in a disc-like alginate slab that was mechanically reinforced by metal grids and was bounded by a microporous membrane on its outer face and a gas-permeable membrane on its inner face. This islet compartment (Fig. 1A) formed part of the outer surface of a disc-shaped structure, named the βAir device (Fig. 1B) that contained a gas chamber with two ports connected to remote subcutaneous access ports through which gas mixtures containing oxygen were replenished daily. The number of islets and the thickness of the islet compartment were maintained within small ranges, and we varied the islet surface density by changing the diameter of the islet compartment (Fig. 1C). With increasing surface density, randomly scattered islets occupied an increasing fraction of the available space but retained their individual identity as single islets or as islets in more concentrated clusters (Fig. 1D). After explantation, islet-alginate slabs retained their initial appearance, intact and without visible damage or cracks in the alginate. With immunohistological examination, islets appeared normal with cells stained for insulin or glucagon as previously described^10^.

### pO_2_ profile in islet-alginate slab

To determine conditions that assured all islets were exposed to an average pO_2_ of about 50 mmHg or more, we used an *in vitro* test system (Fig. 1E) to measure the pO_2_ profile across the slab by introducing an O_2_ electrode to sequentially increasing depths within the slab. Figure 1F shows a representative trace of the microelectrode output (left panel) and the pO_2_ profile within the slab (right panel) following purging of the gas chamber with a gas mixture having a pO_2_ of 304 mmHg while the space above the medium overlying the islet slab was continuously purged with O_2_ and CO_2_, both at a concentration of 40 mmHg and the balance N_2_. After each incremental advance of the electrode, steady state was achieved in less than 30 s, and pO_2_ increased monotonically as distance increased from the top of the islet-containing alginate slab. Measured pO_2_ was maximal near the O_2_-permeable membrane, about 260 mmHg; pO_2_ decreased to a minimum of about 50 mmHg at the outer face of the slab.

The slope of the profile was lower near either boundary than in the center. This likely reflected the heterogeneous distribution of islets in the slab and the large islet size relative to the slab thickness. The center of a spherical islet could not be closer to either slab surface than a distance equal to its radius. Thus, these regions were depleted of islet tissue relative to the core, thereby leading to a lower rate of oxygen consumption and a reduced rate of pO_2_ decrease with distance.

### Minimum required gas chamber pO_2_

A roughly constant quantity of islets was packed in disc-like slabs of successively reduced diameter. With increasing islet surface density, the pO_2_ gradient across the slab increased, resulting in lower values near the host-tissue interface. To compensate for the increased islet density, the gas chamber pO_2_ had to be increased. Because the pO_2_ drop across the oxygen-permeable membrane was estimated to be very small under the conditions of this study, we took the maximum measured pO_2_ in the slab to be the minimum value required in the gas chamber. Measured values of this parameter ranged from 190 to 305 mmHg for islet surface densities of 2,400 to 4,800 IEQ/cm^2^ (Table 1).

**Table 1:** Oxygen partial pressure in the gas chamber.

Islet Density (IEQ/cm^2^)	Minimum pO_2_* (mmHg)	O_2_ partial pressure in the Gas Chamber** (mmHg)	
		Initial pO_2_	Final pO_2_ after 24 hr
2,400	190 ± 31	304	190 ± 22
3,600	229 ± 57	456	280 ± 24
4,800	305 ± 34	570	350 ± 45

^*^About 2,400 IEQ with OCR of 3.4–3.8 pmol/IEQ/min were immobilized at various densities, and a microelectrode was used to measure pO_2_ at different depths in the slab. Minimum pO_2_ is the lowest pO_2_ in the gas required to achieve 50 mmHg at the outer face of the islet-containing alginate slab near the interface with host subcutaneous tissue. N = 3 experiments at each condition.

^**^Devices containing islets at various densities were implanted into diabetic rats. The gas chamber was flushed every 24 hr with 20-ml gas mixtures containing specified oxygen concentrations, 40 mmHg CO_2_, and balance nitrogen. The pO_2_ in the gas chamber was measured at the beginning and after 24 hr, just prior to flushing with the fresh gas mixture. n ≥ 105 different measurements for each islet density as follows: n = 12 rats for 20 days, 10 rats for 25 days, and 3 rats for 35 days, respectively, at islet densities of 2,400, 3,600, and 4,800 IEQ/cm^2^.

The implantable device was designed for O_2_ replenishment to be carried out every 24 hr. During this period, the pO_2_ in the gas chamber decreased as a result of oxygen consumption by islets and escape via diffusion through the encapsulating alginate. Therefore, the initial pO_2_ in the replenishment gas mixture was required to be higher than the measured minimum pO_2_ values summarized in Table 1. To determine the required initial pO_2_, devices loaded with about 2,400 IEQ at various densities were implanted in diabetic rats with different initial gas chamber pO_2_. The gas mixture in each chamber was replenished to its initial level after 24 hours. Just before O_2_ replenishment, the pO_2_ in the gas chamber was measured. Table 1 summarizes the initial pO_2_ and average final pO_2_ after 24 hr. The final value at each islet density equaled or exceeded the minimum gas chamber pO_2_ required, thereby ensuring that the initial pO_2_ levels used were sufficient to maintain the functionality of the islets.

### Maintenance of islet viability

**Figure 2:**
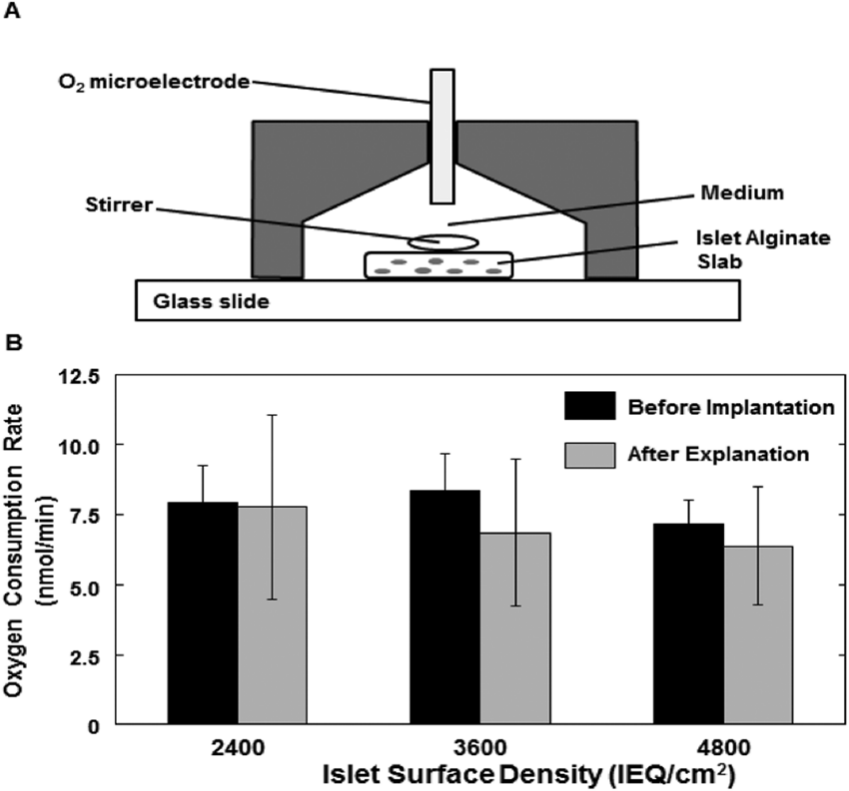
Oxygen consumption rate (OCR) of islets immobilized in alginate slabs. (**A**) Cross-sectional schematic diagram of device for measuring OCR of islets in alginate slabs. A magnetic stirring bar is driven by a magnetic stirrer motor (not shown). The conical chamber is filled with buffer, and pO_2_ is monitored by an oxygen microelectrode. OCR is determined from the rate of change of pO_2_ versus time. Pre-implantation OCR is determined with an islet-alginate slab prepared for this purpose containing about 250 IEQ of the same islets used in the device. The total OCR of the implanted slab is calculated from the measured OCR times the ratio of the number of IEQ actually implanted divided by the number of IEQ in the slab used for OCR measurement. Post-implantation OCR is determined from a sample of the islet-alginate slab carefully removed from the device after explantation. (**B**) OCR recovery after explantation of βAir devices. OCR is plotted before implantation and after explantation. n = 5, 9, and 3 for 2,400, 3,600, and 4,800 IEQ/cm^2^, respectively. The total OCR of the post-implantation slab sample is calculated in a similar way using the number of IEQ in the post-implantation slab sample. Average OCR for naked islet preparations (n = 104) was 340 ± 80 nmol/(min mg DNA), equivalent to 3.4 ± 0.8 pmol/(min IEQ), 1900 ± 400 nmol/(min cm^3^), and 32 ± 7 nmol/(s cm^3^).

Measurements were made in a custom-made stirred cell (Fig. 2A) of islet oxygen consumption rate (OCR) in the alginate slab before implantation and after explantation (Fig. 2B). Recovery of initial OCR was 98, 82, and 89% for 2,400, 3,600, and 4,800 IEQ/cm^2^. For all 17 animals the average OCR recovery was 88%, which indicates that most of the initial viable islet tissue was maintained and very little viable tissue was lost during the implantation period.

### Prediction of minimum chamber pO_2_

**Figure 3:**
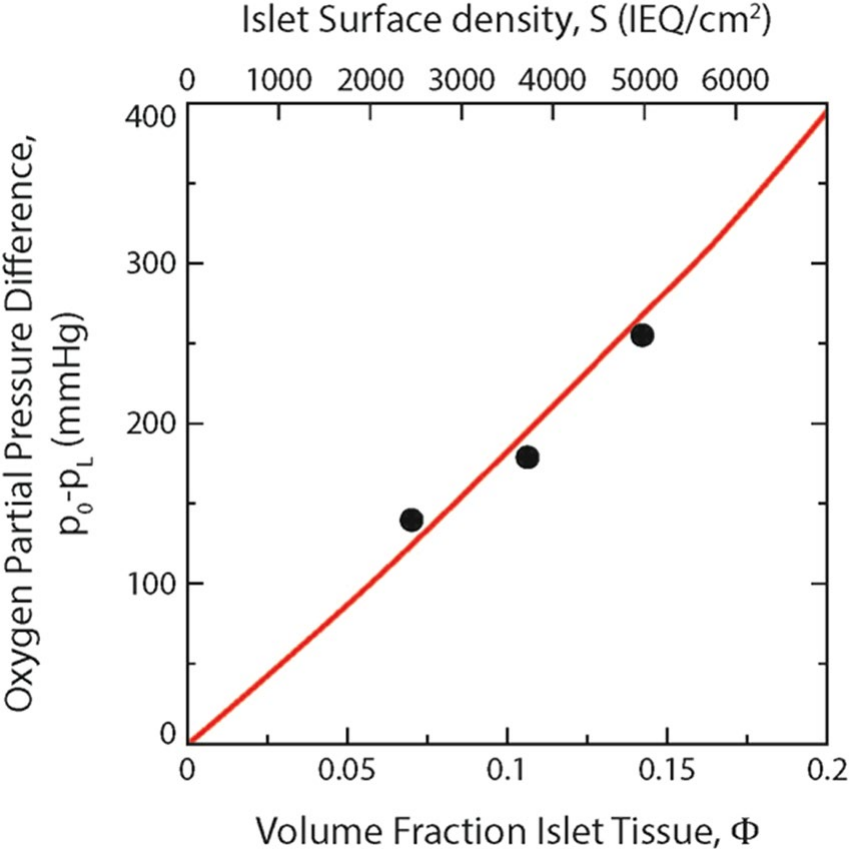
Dependence of required final oxygen partial pressure difference on volume fraction islet tissue or islet surface density. Data points for S = 2,400, 3,600, and 4,800 IEQ/cm^2^ are from Table 1. Curve in red is prediction from Equation (5) calculated with V_max_ = 3.2 × 10^−8^ mol/cm^3^ s, L = 600 μm, p_L_ = 50 mmHg, and other parameters specified in Methods.

A mathematical model was developed by use of a steady state mass balance equating the oxygen diffusion rate to the oxygen consumption rate in a differential slice of the slab in order to relate the design and operating parameters of the device. The minimum required oxygen partial pressure driving force, which is the difference between values in the gas chamber and at the host tissue interface, is plotted versus the islet volume fraction in the slab or, equivalently, the islet surface density, in Fig. 3. The prediction of the model agreed well with the experimental data.

### Maintenance of normoglycemia

**Figure 4:**
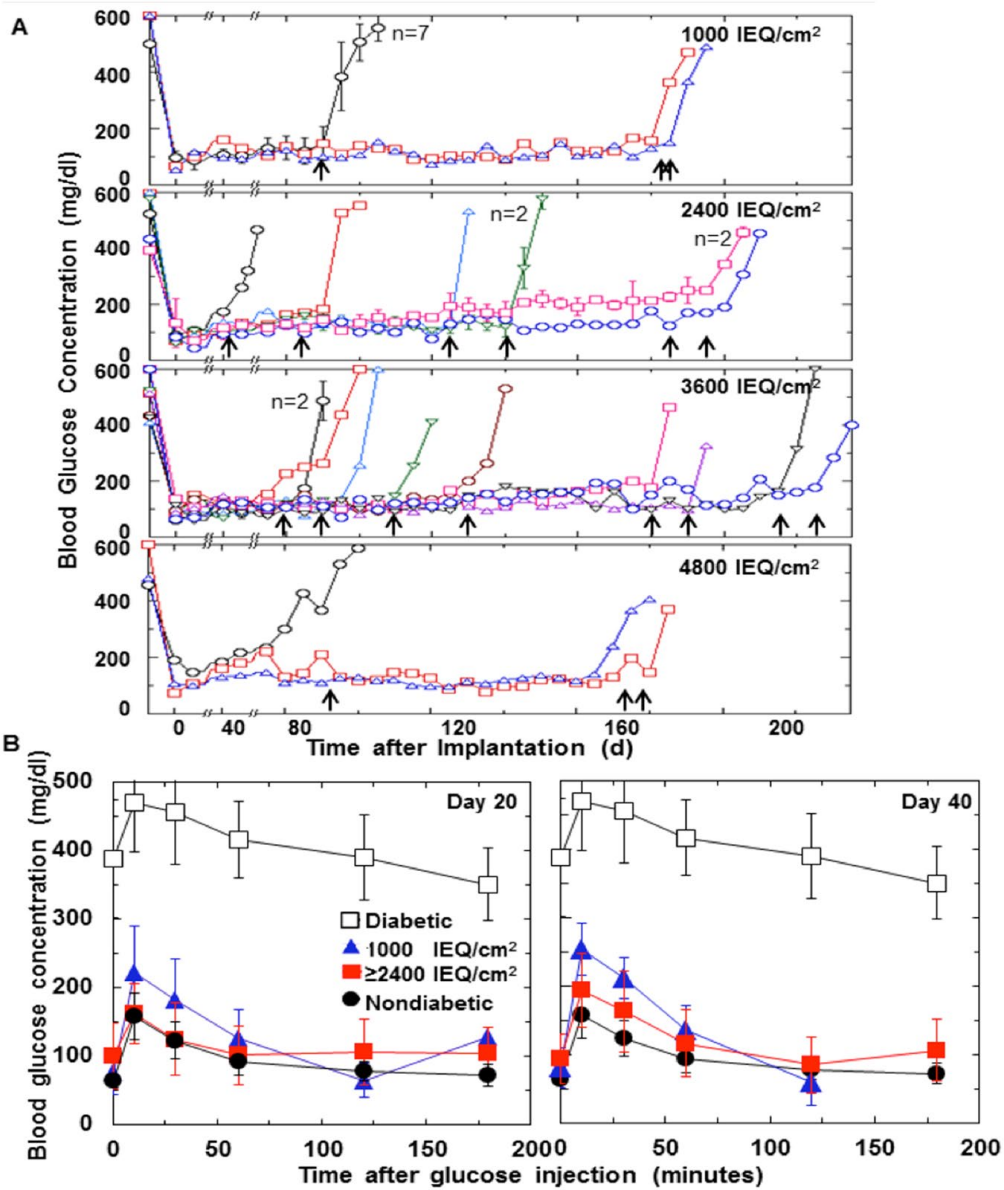
Long-term function of implanted devices with islets encapsulated at various surface densities. (**A**) Blood glucose concentration plotted versus time after implantation. βAir devices implanted with about 2,400 IEQ at surface densities of about 1,000, 2,400, 3,600, and 4,800 IEQ/cm^2^ into streptozotocin-induced diabetic rats. Data from devices with 1,000 IEQ/cm^2^, which were replenished with atmospheric air, are from an earlier study^10^ and are shown for comparison. Data for other devices, which were replenished with gas mixtures having initial pO_2_ shown in Table 1, are taken from the experiments plotted in the upper right domain of Fig. 5A, for which IEQ ≥ 7350 and OCR ≥ 2425. Blood glucose concentration for each symbol represents mean of non-fasting measurements over 5 d taken at 7 am and 4 pm each day (total of 10 measurement for each animal). On day 0, insulin sustained-release capsules were removed. (Data for blood glucose immediately after implantation of these capsules not shown). Thirty devices electively explanted from 42 to 229 d after implantation, indicated by arrows, all but one of which were normoglycemic at time of explantation. n = 2 or higher designates multiple devices treated identically, the results of which were averaged. (**B**) Intravenous glucose tolerance test (IVGTT) performed on day 20 and day 40 following implantation. Data summarized for diabetic rats (n = 28), non-diabetic rats (n = 52), and diabetic rats having device implanted with different surface densities (n = 66). Latter group included all animals that were normoglycemic for 8 weeks or more. IVGTT data plotted for animals having devices with about 1,000 IEQ/cm^2^ and for combined data for devices with 2,400, 3,600, and 4,800 IEQ/cm^2^. For surface densities of about 1,000 IEQ/cm^2^, day20: 0 to 120 min (n = 9), 0 to 180 min (n = 1); day 40: 0 to 120 min (n = 9), 0 to 180 min (n = 0). For 2,400, 3,600, and 4,800 IEQ/cm^2^, respectively, 0 to 180 min, day 20: n = 27, 19, and 8; day 40: n = 26, 15, and 5.

Devices containing roughly 2,400 IEQ at four different surface densities were implanted in 137 rats. Islet function in implanted devices was assessed by monitoring glucose homeostasis. Almost all animals remained normoglycemic for different periods of time including 66 that remained normoglycemic for more than 8 weeks. Blood glucose concentration from devices in selected animals that were explanted for subsequent tests are plotted in Fig. 4A. One device was removed after the animal became hyperglycemic at 42 days post-implantation. All other animals remained normoglycemic until device explantation from 78 to 238 days following implantation, after which blood glucose concentration rapidly rose to hyperglycemic levels. Normoglycemic periods of 6 months or more were observed in some animals with all surface densities.

### Intravenous glucose tolerance tests

IVGTT data were obtained from 66 animals with implanted devices (Fig. 4B). The results when measured on day 20 and again on day 40 following implantation (Fig. 4B) were very similar to that of normal nondiabetic animals. There was no systematic significant difference between data for nondiabetic animals and animals with implanted devices having surface densities of 2,400, 3,600, and 4,800 IEQ/cm^2^. There was a significant increase in blood glucose concentration at short times for animals with 1,000 IEQ/cm^2^.

### Correlation between normoglycemia time and islet quality parameters

**Figure 5:**
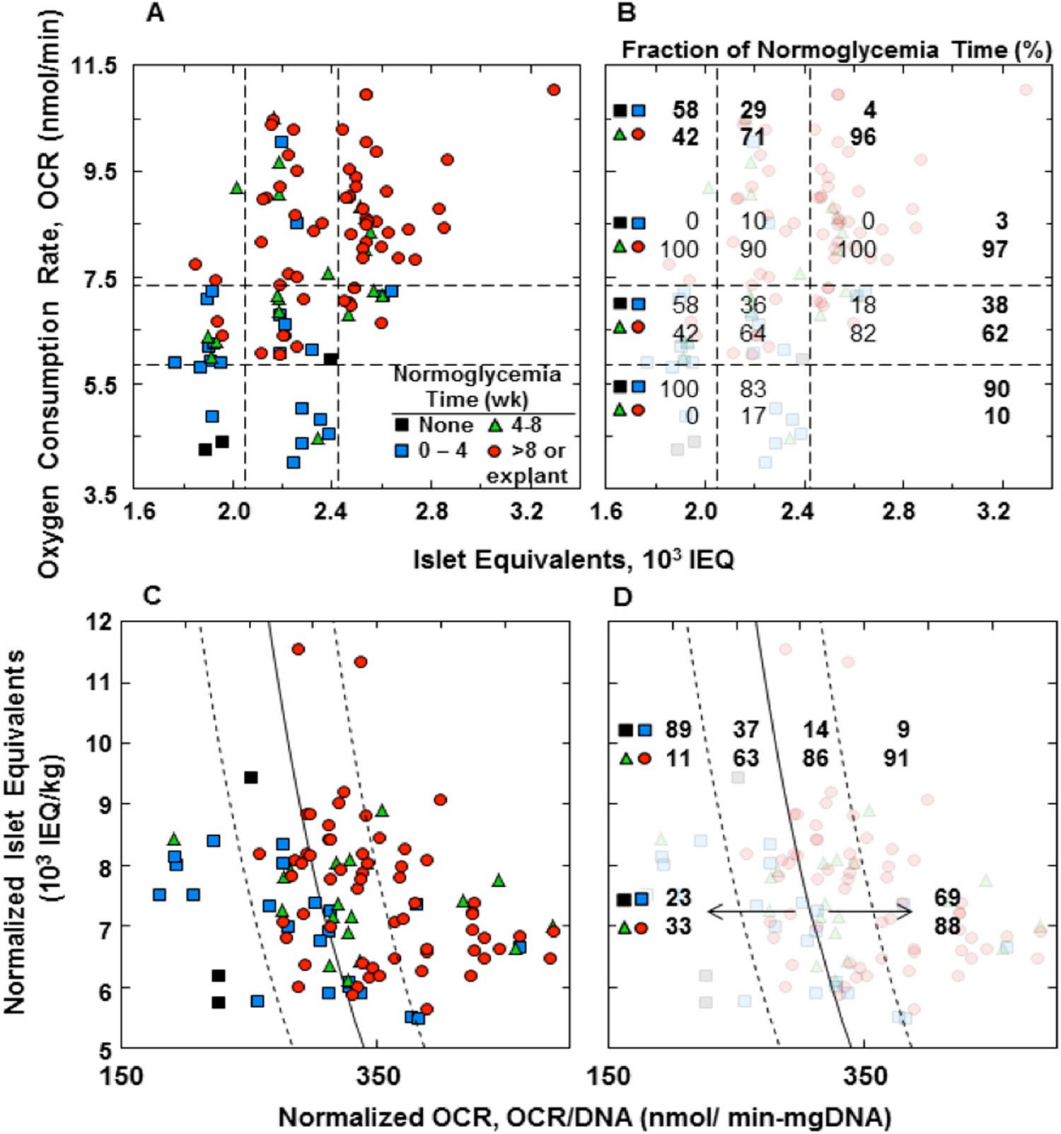
Relationship between normoglycemia time and islet parameters of animals for which pre-implant data is available. Time period during which normoglycemia was maintained: (1) none, (2) >0–4 wk, (3) 4–8 wk, and (4) 8 wk or explantation while still normoglycemic. (**A**) Datum point for each animal plotted at its corresponding OCR and number of IEQ. Data field is segregated into nine regions defined by IEQ ≤ 2050, 2050 ≤ IEQ ≤ 2425, and 2425 ≤ IEQ; OCR ≤ 5850, 5850 ≤ OCR ≤ 7350, and 7350 ≤ OCR. (**B**) Fraction of normoglycemia times <4 wk (top two symbols) and >4 wk (bottom two symbols) indicated within each region. Average for each row and column is also tabulated in bold. (**C**) Same data as in (**A**) plotted on coordinates of IEQ/kg and OCR/DNA. DNA content was calculated using 1,560 cells/IEQ^13^ and 6.5 mg DNA/cell^17^. Solid curve was fitted to data for naked transplantation of rat islets into mice^18^, given by log_10_ y = ax + b, where y = IEQ/kg, x = OCR/DNA in nmol/min mg DNA, a = 0.00515 DNA/OCR, and b = 5.45. Dashed lines to left and right of fitted curve are 68% confidence limits associated with fitted curve. (**D**) Same as (**C**), with the fraction of normoglycemia time <4 wk (top) and >4 wk (bottom) indicated for each region.

Normoglycemia time in animals with implanted devices ranged from a minimum of none to a maximum of 229 days after elective explantation from an animal still normoglycemic. We hypothesized that variations in the quality of islet preparations immobilized in the devices accounted for at least part of this variation. Animals were assigned to groups having normoglycemia times of (1) none, (2) >0 to 4 wk, (3) 4 to 8 wk, or (4) >8 wk or time of explantation (always greater than 8 wk). The datum point for each animal is plotted on coordinates of number of IEQ versus pre-implantation OCR of the immobilized islets (Fig. 5A,B), and each axis is divided into three domains. Normoglycemia time increased with increasing IEQ and increasing OCR. The fraction of animals displaying long term (>4 wk) normoglycemia was 100% in the upper right domain and 0% at the lower left. Nearly 100% was observed for IEQ > 2,425; the same was true for OCR > 7,350 pmol/min. We normalized the data in terms of IEQ/kg and OCR/DNA (Fig. 5C,D) for comparison with results from a previous study^18^ with naked rat islets transplanted in mice, shown by the solid curve, which represents the predicted marginal islet mass based upon these quality parameters. The fitted curve from the previous study agreed with data from the current study. To the right of the curve, 69% of the animals were normoglycemic for >8 wk and 88% for >4 wk. To the left of the curve, only 33% were normoglycemic >4 wk, and 6% never attained normoglycemia.

Islet insulin content pre-implantation was also measured in all experiments. In contrast to IEQ and OCR, insulin content was not useful as an islet quality parameter. The Pearson correlation coefficient between mean blood glucose for 29–32 d after implantation and total insulin content was 0.06, indicating no correlation. Average normalized insulin content of 75 ± 40 mg/IEQ for implantations in the upper right domain in Fig. 5A was not significantly different from the value of 74 ± 30 mg/IEQ for implantations in all of the other eight domains, indicating that the parameter was not predictive of transplantation outcome in this study.

## Discussion

A native pancreatic islet is well vascularized, which results in nearly uniform oxygen concentration of 38–40 mmHg throughout the islet^18^. In isolated islets, O_2_ must diffuse from the surface into the islet core; therefore, a higher concentration of O_2_ must be supplied on the surface of an islet. The subcutis (SC) is an attractive site for transplantation because of the simplicity of implantation. However, the O_2_ level in the SC is only about 40 mmHg, which is insufficient to fully support islets above about 100 µm in diameter^19^ and will lead to deleterious effects on insulin secretion^4,15^ from human islets that typically average about 150 µm diameter. Attempts to implant macroencapsulated islets that rely on oxygen supply from the surrounding tissue eventually fail to achieve prolonged satisfactory function except for one report with a monolayer device^20^. Encapsulation of islets exacerbates oxygen supply limitations, which leads to degradation of islet glucose-dependent insulin secretory capability and loss of viability.

We have used a retrievable, macroencapsulated planar diffusion chamber for subcutaneous islet implantation in a rat in which exogenous oxygen is supplied by diffusion from a gas chamber to one side of an alginate-islet slab. We explored the relationship between increased surface density of encapsulated islets and the initial pO_2_ required in the gas chamber to ensure that at the end of 24 hr, just prior to replenishment, the minimum pO_2_, at the surface of islets nearest the host-device interface would be about 50 mmHg or greater, thereby ensuring that the islets remain fully viable and functional.

We made *in vitro* measurements of the pO_2_ profile in the alginate-islets slab and of pO_2_ changes in the gas chamber to estimate the approximate required pO_2_ in the gas mixture (Table 1). For example, to support the highest density tested of 4,800 IEQ/cm^2^, a minimum pO_2_ of about 305 mmHg was needed. An initial gas chamber pO_2_ of 570 mmHg dropped after 24 hr to about 350 mmHg. Thus, an initial pO_2_ of 570 mmHg was sufficient to support all islets at a density of 4,800 IEQ/cm^2^. Because the minimum pO_2_ levels required in the gas chamber are much higher than that in the microvasculature, this result could not have been achieved by any other method that relies upon the local oxygen supply through the bloodstream, even if extensive neovascularization surrounding the implant had been achieved.

Nearly all of the viable islets initially implanted in the device retained their viability throughout the duration of these experiments, even at the highest density of 4,800 IEQ/cm^2^, as demonstrated by high OCR recovery at explantation (Fig. 2). The small reduction in OCR for explanted islets may have resulted in part, from normal islet turnover^21^. To our knowledge, this is the first report of quantitative data demonstrating long-term maintenance of islet viability following naked or encapsulated transplantation. We assumed that total OCR is a measure of the amount of viable tissue, but we cannot conclude that OCR/cell remained constant or perhaps increased, without data for the number of cells in each slab tested^22^.

We previously employed a complex, detailed numerical model to explore the interaction of oxygen diffusion and consumption in assemblages of spherical islets dispersed in an alginate slab^17^ in order to predict conditions required to maintain islet viability and function. Here we employed a simpler model amenable to analytical solution that treated the slab as a homogenous medium characterized by effective properties derived using those of the individual phases. To our knowledge, this is the first attempt to predict the behavior of experimental data obtained with a real encapsulation system. The remarkable agreement between prediction and data (Fig. 3) validates the mechanism and hypothesis upon which development of the device is based as well as the parameter values employed. Furthermore, the equations that result from the analytical solution, which can also be derived for all other basic geometries, such as cylinders and spheres, provide a simple design framework that relates the oxygen partial pressure driving force, device geometry and dimensions, islet tissue loading (in terms of islet volume fraction or surface density), islet OCR, and oxygen permeability of islets and alginate. This framework provides a powerful tool for translation to the clinic of devices with new designs incorporating (1) larger amounts of islet tissue, (2) islets of different species, such as porcine, and (3) differentiated pluripotent cells that have different OCR and islet secretion properties.

Most devices containing 2,400 IEQ at densities up to 4,800 IEQ/cm^2^ implanted into diabetic rats maintained normal fasting blood glucose for 4 wk or more. In animals normoglycemic for 8 wk or more or until elective termination of the experiments up to 229 days after implantation (Fig. 2A), no detectable delay in the near-normal IVGTT was observed (Fig. 2B), demonstrating fast response of the device implant in the subcutis of a rat. Normalization of IVGTT by itself does not prove that the islets are functioning normally. As long as the tissues, in particular the liver and muscle, are well insulinized, glucose can be controlled even though the timing of insulin release from encapsulated islets is abnormal^23^. Nonetheless, islets immobilized at very high densities in the alginate slab survived in the βAir device for a long period of time without deterioration of glucose control and without apparent damage from exposure to pO_2_ levels greater than physiologic.

Normoglycemia time varied widely between different implantations under otherwise identical conditions. This variation correlated with islet quality parameters IEQ and OCR^24^, which emphasizes the importance of initial islet quality in determining the outcome of each transplantation. When expressed as normalized parameters IEQ/kg body weight and OCR/DNA, our results were comparable to an earlier study with naked rat islets transplanted into mice^25^. Scatter along the OCR/DNA axis may have been amplified because DNA was calculated from IEQ measurements made visually, which themselves are subject to significant scatter^26^. Healthier islet preparations with higher OCR survived for long periods with little loss of viable tissue while maintaining normoglycemia. Under the same implantation conditions, islets with reduced OCR, which indicates mitochondrial damage and possibly continued ongoing cell death processes at the time of transplantation, resulted in shorter periods of normoglycemia (Fig. 5). The poorer quality islet preparations did not improve when implanted *in vivo* in the device. In the field of organ preservation, use of perfusion circuits to mimic healthy physiologic conditions have been suggested as a means to allow organs to recover from cellular stress and tissue injury during donor death and organ procurement^27^. Our findings suggest that islets isolated using current methods would not benefit from such an approach.

An important finding of this study is that the size of a device for implantation in humans can be substantially reduced. Consequently, for example, a dose of 250,000 IEQ could be supported under these conditions in a device having about 50 cm^2^ surface area for supply of O_2_ from the gas chamber. By mating two islet compartments back to-back with a sandwiched gas chamber, the surface area required for islet support could be farther reduced to 25 cm^2^, which is achieved by a disk with a diameter of 5.6 cm or about 2 ¼ in. A dose of 500,000 IEQ would require two such devices or a single device with 8.0 cm diameter. Such size reduction would make implantation in humans more feasible.

In addition to reduction of implanted device size, these findings suggest substantial benefits in comparison to naked islet transplantation. Human islet transplantation requires about 10,000 or more IEQ/kg body weight, often supplied from two or more cadaveric donors^28^. Studies in mice^29^ and in clinical islet transplantation^30,31^ indicate that only a fraction of the islet dose survives the implantation and early engraftment period. In contrast, our results demonstrate that enhanced *in situ* supply of exogenous oxygen directly to islets encapsulated at high densities can maintain viability and function of the islets with only a small loss over long periods of time. By eliminating the substantial loss of viability and function currently experienced, the limited supply of islets can be used much more efficiently, and human preparations normally discarded because of insufficient numbers of islets might be used fruitfully.

Minimization of islet cell death may also enhance the immunoprotection provided by encapsulated alginate in the case of stimulation of the indirect pathway, which mediates rejection of xenografts and may also be operative with allografts^32^. Host antigen-presenting cells take up graft proteins and peptides released from the encapsulated cells and present donor-derived peptides on host MHC molecules to host T-cells. The extracellular domain of many cell surface and membrane-anchored proteins is normally released by proteolysis to produce shed antigen^33,34^, some of which may be small enough to permeate across an immunobarrier material such as alginate hydrogel. The rate of antigen release is sharply increased when peptides are released from the interior of dying cells^35–37^. Hence, the presence of dying cells in encapsulated islets may generate a large immunogenic stimulus, especially with xenografts, which triggers the indirect pathway and ultimately leads to attack by agents released from activated immune cells that form a florid response around the implant. By maintaining the viability of virtually all islets encapsulated in the βAir device, development of this immunogenic stimulus is prevented, and immunoisolation can be attained. This mechanism may have played an important role in the previous demonstrations with the device of successful xenotransplantation in minipigs^12^ and allotransplantation in a human^13^, both without immunosuppression.

Here we show long-term quantitative preservation of islet viability and function after transplantation in a retrievable macroencapsulation device containing islets in an alginate slab bounded on one face by supraphysiological levels of oxygen. Islet densities as high as 4,800 IEQ/cm^2^ are maintained viable and functional with initial gas chamber pO_2_ of 570 mmHg. To our knowledge, this is the first successful demonstration of implantation with such a high tissue density. Use of a novel OCR assay before implantation and after explantation shows nearly 90% preservation of islet viability. This is the first report of quantitative preservation of islet viability with any encapsulation system. The same benefits will accrue to xenogeneic islets and to islet cells derived by differentiation of stem cells. The results reported here indicate that the enhanced oxygen supply in the βAir device provides a suitable approach for treating diabetes with encapsulated islets and for other cell therapies in humans. In addition, the simple mathematical model provides a framework for design of clinical devices based on device operating conditions and tissue properties.

## Materials and Methods

### Animals, diabetes induction, and pre-treatment

Lewis rats (260–280 g) were purchased from Harlan (Rehovot, Israel), and diabetes was induced by a single intravenous infusion of 85 mg/Kg body weight of Streptozotocin (STZ; Sigma, Israel) as previously described^10^. Animals had free access to food at all times and were considered diabetic when non-fasting blood glucose exceeded 450 mg/dl for 4 consecutive days or more. To prepare the diabetic animals for device implantation in a non-stressing, close-to-normal blood glucose environment, 1.5 capsules of a sustained release insulin implant (Linplant, LinShin, Toronto, Canada) were inserted under the skin of the diabetic animals, which were considered ready for implantation of the device when their non-fasted blood glucose was under 250 mg/dL for 3 consecutive days or more. The sustained-release insulin capsules were removed 48 hr after device implantation, leaving the encapsulation device as the only source for insulin. The efficacy of glycemic control was followed after implantation by assessing the functionality of the islets in the device through twice daily measurements of non-fasting blood glucose concentration. Animals were sedated, blood samples were collected from the tail, and glucose levels were measured by glucometer (Accu-Chek sensor, Roche Diagnostics GmbH). Blood glucose concentration ≤200 mg/dl was deemed to be normoglycemic. Intravenous glucose tolerance tests (IVGTT) were performed 21 and 42 days post-transplantation as follows: animals were fasted overnight. On the following morning, 1 ml of 0.7 M glucose solution was infused within 10–15 sec (dose of 500 mg/kg BW), and blood glucose samples were collected for measurement before infusion and at 10, 30, 60, 120 and 180 min following glucose infusion. Selected devices were electively explanted from normoglycemic animals for further study at times ranging from 42 to 238 days post-transplantation.

### Islet isolation and culture

Pancreata from 9 to 10-week old male Lewis rats weighing 260–280 g underwent collagenase digestion following a standard procedure with slight modification, as described previously^10^. Briefly, each pancreas was infused with 10 ml enzymatic digestive blend containing 15 PZ units of collagenase NB8 (Serva, Heidelberg, Germany) and 1 mg/ml bovine DNAse (Sigma, Israel) dissolved in HBSS solution (Bet-HaEmek, Israel) for 14 min. Islets were purified on discontinuous Histopaque gradient (1.119/1.100/1.077 in RPMI) run for 20 min at 1,750 × g and 6 °C, washed twice, and cultured in complete CR medium (1:1, CMRL:RPMI medium, Bet HaEmek, Israel), supplemented with 10% fetal bovine serum (Bet-HaEmek, Israel) for 1 wk prior to being integrated in implantable devices. Measurement of islet size and number in a preparation, from which islet volume and number of IEQ were determined, was carried out by visual counting as previously described^10^. Insulin content of islets was measured with acid/ethanol extraction and ELISA (ThermoFisher Scientific). Islet purity estimated visually was always greater than 95%. Naked islet viability, assessed as the fraction of green cells measured with the Acridine Orange/Propidium Iodide membrane integrity assay was always greater than 95%.

### The βAir device

The subcutaneously-implantable device, named βAir^10^, had an external disc-shaped housing made of clinical grade polyether ether ketone (PEEK Optima LT1R40; Invibio, Lancashire, UK) with a diameter of 31.3 mm and thickness of 7 mm (Fig. 1). The device consisted of three major components: (1) The islet compartment contained about 2,400 IEQ embedded in 500 to 600-µm thick ultrapure high guluronic acid alginate, reinforced with 100-µm thick stainless steel grids having about 80% fractional open area (top grid, Fig. 1A, inset, Suron, Maagan Michael, Israel), glued to the PEEK housing with medical epoxy adhesive (Epotek 301–2, Epoxy Technology Inc., Billerica, MA, USA). Mechanical support was provided by the bottom grid, identical to the top grid, which was placed under the gas permeable membrane and reinforced by PEEK mechanical supports (Fig. 1B). To vary islet surface density, 2,400 IEQ were immobilized in a slab with a diameter of 11.3, 9.3, or 8.0 mm, resulting in densities of 2,400, 3,600, or 4,800 IEQ/cm^2^ en face surface area for oxygen transport, respectively (Fig. 1B,C). (2) The gas chamber (3-ml volume) was separated from the islet compartment by a 25-µm gas-permeable silicone rubber-teflon membrane (Silon, BMS, Allentown, PA) and contained inlet and outlet gas chamber ports connected by two polyurethane tubes to subcutaneous access ports (Cat. No. PMINO-PU-C70, Instech Solomon, PA) implanted under the skin at a site remote from the device, as previously described^10^. (3) A 25-µm, 0.4-µm pore diameter hydrophilized microporous polytetrafluoroethylene (PTFE) membrane (Biopore, Millipore, Billerica, MA), separated the islet module from host tissue and protected the islets from the cellular part of the immune system. Devices with a diameter of 18 mm were used to give islet densities of 1,000 IEQ/cm^2^. Some of these devices made use of an earlier, less effective design^10^ and the results are provided here selectively for comparison.

### Device assembly

An average dose of 2,400 ± 200 IEQ (range 1,700–3,300 IEQ) was collected by 5-min sedimentation. The pellet was gently mixed with 2.2% (w/v) ultrapure high-guluronic acid (68%) alginate (Pronova UPMVG, Novamatrix; Sandvika, Norway). The mixture was placed in the islet module compartment and spread through the openings of the top grid (Fig. 1A, inset) with the tip of a long-nosed Pasteur pipette. The microporous PTFE (Biopore) membrane was then fixed onto the device using a Viton O-Ring (hardness 75 Shore and outer diameter 27 mm, McMaster Carr, Aurora, OH) and sealed to the plastic housing with medical silicone adhesive (MED 2000, Polytek Easton, PA). The alginate was cross-linked by applying a flat sintered glass disc (Pyrex, UK) saturated with strontium chloride dissolved in RPMI medium for a final concentration of 70 mM. The device and sintered glass were immersed in the RPMI-strontium medium for 16 min, resulting in a 500- to 600-µm thick coin-like slab. The thickness variations originated with variation in glue thickness. The device was washed for an additional 5 min at 37 °C in complete CR medium (Beit HaEmek, Israel). Fully fabricated devices were washed in complete CR medium at 37 °C with agitation for 2 h before implantation.

### Device implantation

All animal experiments were performed in strict accordance with the Institutional Animal Care and Use Committee Guidebook. The study was approved by The Council for Experiments on Animal Subjects, Ministry of Health, Israel (Permit No. IL05-05-012). All efforts were made to minimize animal suffering. Rats were anesthetized by intraperitoneal injection of 90 mg/Kg Ketamine and 10 mg/Kg Xylazine followed by isoflurane inhalation. A 3-cm incision was made for the device on the dorsal skin, and muscles were separated from the hypodermis. A second incision was made in the skin between the shoulder blades, and two channels connecting this site with the device implantation site were created by traversing 3-mm wide stainless steel needles under the skin. The device was inserted under the dorsal skin incision with the islet module facing the fascia, and the gas chamber ports were connected to the remote subcutaneous access ports. The skin was sutured and fixed with a tissue adhesive (Histoacryl, Tufflingen, Germany). Devices were implanted into 141 animals. Data was discarded from four animals due to mechanical problems with the device.

### Gas mixture replacement

Every 24 h the animal was sedated with isoflurane inhalation. A 27G needle was inserted into each of the two implanted access ports, and the gas chamber was purged with 20 ml (about 6.7 chamber volumes) of gas mixture containing the specified pO_2_, 40 mmHg CO_2,_ and balance N_2_. Final total pressure in the gas chamber was equal to ambient atmospheric pressure. To obtain the different oxygen mixtures, prefilled cylinders were used (Maxima, Israel).

### Islet oxygen consumption rate (OCR)

OCR of naked islets was measured as described previously^10^. Measurements were also made with an immobilized aliquot of 250 IEQ from the preparation to be transplanted in the device and with the islet alginate slab after device explantation. The preimplant islet sample was immobilized in 30 μL of high guluronic acid alginate shaped as a coin with a thickness of 500 μm and diameter of 8.7 mm. After elective explantation of a device, the alginate slab containing the islets was carefully removed. The number of IEQ (≥200 islets) in a small defined surface area was quickly determined by manual counting under a microscope. The slab having the largest diameter was cut into a smaller piece that fit into the measurement chamber. The pre- or post-implant slab was placed on a glass slide, a 5-mm diameter magnetic stirrer bar was placed on top of the slab, and the assembly was covered with a conical OCR measurement chamber (Fig. 2). The conical chamber was filed with 1:1, CMRL:RPMI medium containing 1% (v/v) fetal bovine serum to a final volume of 620 µl. The chamber was equipped with Clark-type oxygen electrode of 500-µm diameter connected to a picoammeter controller (Cat No. PA2000, Unisense, Arhaus, Denmark). The O_2_ measurement chamber was placed within a Perspex box with the air temperature maintained at 37 ± 1 °C using a temperature control unit (Eurotherm 808; Eurotherm Worthing, UK). The stirring speed was increased until measured OCR did not change (about 70 rpm), thereby assuring minimal effects associated with mass transfer boundary layers around the islets and the O_2_ electrode. No damage to the alginate slab or the islets was observed as assessed by islet and slab morphology and stable OCR readings. The electrode was calibrated using medium equilibrated with gas containing zero or ambient air oxygen concentrations. The O_2_ level in both phases are reported here as oxygen partial pressure pO2, in units of mmHg, which is related to oxygen concentration c by the equation c = αpO_2_, where α is the Bunsen solubility coefficient, 1.34 × 10^−9^ mole/(cm^3^mmHg) for oxygen in medium at 37 °C. Consequently, for example, at steady-state ambient O_2_ partial pressure of 160 mmHg (21% O_2_, 1 atm), dissolved O_2_ concentration is 215 µM in the medium at 37 °C. As a result of the O_2_ consumption by the islets, the O_2_ concentration in the medium within the conical measurement chamber decreased with time from its initial value in equilibrium with ambient air. The data for O_2_ concentration versus time was fitted by linear regression, and the slope was used to estimate OCR of the islets^38^. Data was used between 160 and 80 mmHg, which yielded the highest slope. Estimation of OCR by this method required that the pO_2_ profiles within the slab were in quasi-steady state and that the pO2 difference between the medium and the slab interior was small. Approximate theoretical analysis similar to that previously used for OCR measurement with microencapsulated islets^39^ indicated that these conditions were met for the measurements made in this study.

### Oxygen gas measurements

To measure O_2_ concentration in the gas chamber within the implanted devices, a 27G needle connected to 1.0 ml syringe was inserted into one of the implanted subcutaneous access ports, and a 250-µl sample was taken from the gas chamber 24 hr after the last O_2_ replenishment and injected into the conical measurement chamber. The change in the electrode measurement was used to calculate the oxygen concentration in the sample from the gas chamber. The O_2_ electrode was calibrated with gas mixtures having pO_2_ of zero (pure N_2_), 160 mmHg (ambient air), and 304 mmHg.

### Oxygen profile across the islet slab

About 2,400 IEQ were immobilized at various densities as described for βAir device assembly, but without the PTFE (Biopore) membrane and without the metal grid on top. The device was placed in a covered 90 mm Petri-dish, overlain with RPMII medium so as to create a layer of minimal depth on top of the device, and the space above the slab was purged with a gas stream having 40 mmHg O_2_, 40 mmHg CO_2,_ and 680 mmHg N_2_ (Fig. 3), which simulated the gas composition in the subcutis. The gas chamber was purged with oxygen concentrations varying between 152 and 305 mmHg. An O_2_ electrode with a diameter of 500 µm, attached to a micromanipulator, was inserted into the islet-containing slab and advanced at 100 µm increments from the distal side of the islet slab downwards toward the gas permeable membrane. At each step, the O_2_ electrode readings reached a steady-state level before moving to the next step. The entire measurement system was located in a 37 °C chamber. Although the presence of the electrode would disturb the O_2_ field between the surface of the oxygen-permeable membrane and the face of the electrode, the pO_2_ measurement of primary interest here was the minimum value farthest from the gas chamber where the electrode first entered the slab. Under these conditions, the error incurred in determining if the pO_2_ is about 50 mmHg is expected to be small.

### Mathematical model of oxygen diffusion and consumption in islet-alginate slab

We consider a one-dimensional slab of thickness L containing a heterogeneous medium of islets dispersed in alginate. Oxygen concentration c is linearly proportional to oxygen partial pressure p according to c = αp, where α is the Bunsen solubility coefficient. At the slab face next to the gas chamber, x = 0 (the small effect of the Silon membrane is ignored), the oxygen partial pressure is set at p_0_. At the slab surface that interfaces with host tissue, we assume the most conservative case that all oxygen is supplied by the gas chamber, and the interface is taken to be impermeable to oxygen. We set the oxygen partial pressure at this surface, x = L, as p_L_ = 50 mmHg, to be consistent with experimental conditions, in which case the oxygen consumption rate can be assumed constant at V_max_^40^. Under these conditions, a steady state oxygen mass balance equating the rate of diffusion to the rate of consumption across a differentiated slice of the slab can be written as

1 $${({\rm{\alpha }}{\rm{D}})}_{{\rm{eff}}}\frac{{{\rm{d}}}^{2}{\rm{p}}}{{{\rm{dx}}}^{2}}={{\rm{V}}}_{{\rm{\max }}}\varphi $$

where (αD)_eff_ is the effective permeability of oxygen in the slab given by

2 $$\frac{{({\rm{\alpha }}{\rm{D}})}_{{\rm{eff}}}}{{({\rm{\alpha }}{\rm{D}})}_{{\rm{c}}}}=\frac{2-2\varphi +\rho (1+2\varphi )}{2+\varphi +\rho (1-\varphi )}$$

*ϕ* is the volume fraction of the dispersed phase, and *ρ* is a permeability ratio

3 $$\rho =\frac{{({\rm{\alpha }}{\rm{D}})}_{{\rm{d}}}}{{({\rm{\alpha }}{\rm{D}})}_{{\rm{c}}}}$$

The solution is given by^41^

4 $$\frac{{\rm{p}}({\rm{x}})}{{{\rm{p}}}_{0}}=\frac{{{\rm{V}}}_{{\rm{\max }}}\varphi {{\rm{L}}}^{2}}{{({\rm{\alpha }}{\rm{D}})}_{{\rm{eff}}}{{\rm{p}}}_{0}}\{\frac{1}{2}{(\frac{{\rm{x}}}{{\rm{L}}})}^{2}-(\frac{{\rm{x}}}{{\rm{L}}})\}+1$$

Evaluating Equation (4) at x = L yields a relation between the required oxygen partial pressure difference and the other variables.

5 $${{\rm{p}}}_{0}-{{\rm{p}}}_{{\rm{L}}}=\frac{{{\rm{V}}}_{{\rm{\max }}}\varphi {{\rm{L}}}^{2}}{{2({\rm{\alpha }}{\rm{D}})}_{{\rm{eff}}}}$$

or an expression for the maximum thickness possible for prescribed operating conditions.

6 $${\rm{L}}={[\frac{{2({\rm{p}}}_{0}-{{\rm{p}}}_{{\rm{L}}}{)({\rm{\alpha }}{\rm{D}})}_{{\rm{eff}}}}{{{\rm{V}}}_{{\rm{\max }}}\varphi }]}^{1/2}$$

Noting that islet surface density S can be expressed by

7 $${\rm{S}}\,\,\,=\,\,\,\frac{{\rm{L}}{\varphi }}{{{\rm{V}}}_{{\rm{IE}}}}$$

where V_IE_ is the volume of an IEQ (1.77 × 10^−6^ cm^3^/IEQ), Equation (6) can be rearranged to express the maximum surface density attainable

8 $${\rm{S}}=\frac{1}{1.77\times {10}^{-6}}={[\frac{{2({\rm{p}}}_{0}-{{\rm{p}}}_{{\rm{L}}}{)({\rm{\alpha }}{\rm{D}})}_{{\rm{eff}}}{\varphi }}{{{\rm{V}}}_{{\rm{\max }}}}]}^{1/2}$$

### Statistics

Data are expressed as mean + standard deviation. Statistical significance (p < 0.05) was determined by Student’s t-test.

## References

[1] Scharp DW, Marchetti P (2014). Encapsulated islets for diabetes therapy: History, current progress, and critical issues requiring solution. Adv. Drug Delivery Rev..

[2] Colton CK (2014). Oxygen supply to encapsulated therapeutic cells. Adv. Drug Delivery Rev..

[3] Tannock IF (1972). Oxygen diffusion and the distribution of cellular radiosensitivity in tumors. Br. J. Radiol..

[4] de Groot M (2003). Response of encapsulated rat pancreatic islets to hypoxia. Cell Trans..

[5] Wu H (1999). *In situ* electrochemical oxygen generation with an immunoisolation device. Ann. N.Y. Acad. Sci..

[6] Avgoustiniatos ES, Colton CK (1997). Effect of external oxygen mass transfer resistances on viability of immunoisolated tissue. In Bioartificial Organs (Ann. N.Y. Acad. Sci.).

[7] Bloch K (2006). Photosynthetic oxygen generator for bioartificial pancreas. Tissue Eng..

[8] Evron Y (2015). Oxygen supply by photosynthesis to an implantable islet cell device. Horm. Metab. Res..

[9] Pedraza E, Coronel M, Fraker CA, Ricordi C, Stabler CL (2012). Preventing hypoxia-induced cell death in beta cells and islets via hydrolytically activated, oxygen-generating biomaterials. Proc. Natl. Acad. Sci. USA.

[10] Barkai U (2013). Enhanced oxygen supply improves islet viability in a new bioartificial pancreas. Cell Transpl..

[11] Ludwig B (2012). Improvement of islet function in a bioartificial pancreas by enhanced oxygen supply and growth hormone releasing hormone agonist. Proc. Natl. Acad. Sci. USA.

[12] Neufeld T (2013). The efficacy of an immunoisolating membrane system for islet xenotransplantation in minipigs. PLoS ONE.

[13] Ludwig B (2013). Transplantation of human islets without immunosuppression. Proc. Natl. Acad. Sci. USA.

[14] Pisana A (2010). Quantitative analysis of cell composition and purity of human pancreatic islet preparations. Lab. Invest..

[15] Dionne KE, Colton CK, Yarmush ML (1993). Effect of hypoxia on insulin secretion by isolated rat and canine islets of Langerhans. Diabetes.

[16] Colton CK (1995). Implantable biohybrid artificial organs. Cell Transp..

[17] Johnson AS, Fisher RJ, Weir GC, Colton CK (2009). Oxygen consumption and diffusion in assemblage of respiring spheres: Performance enhancement of a bioartificial pancreas. Chem. Engineering Sci..

[18] Carlsson PO, Palm F, Andersson A, Liss P (2001). Markedly decreased oxygen tension in transplanted rat pancreatic islets irrespective of the implantation Site. Diabetes.

[19] Lewis, A. S., Fisher, R. J., Weir, C. G. & Colton, C. K. Improving oxygen supply to encapsulated cells and islets. In *The Bioartificial Pancreas and Other Biohybrid Therapies* (eds Halle, J.-P., De Vos, P. & Rosenberg, L.) 205–241 (Transworld Research Network, 2009).

[20] Dufrane D, Goebbels RM, Gianello P (2010). Alginate macroencapsulation of pig islets allows correction of streptozotocin-induced diabetes in primates up to 6 months without immunosuppression. Transplantation.

[21] Bonner-Weir S (2001). β-cell turnover: Its assessment and implications. Diabetes.

[22] O’Sullivan ES (2010). Rat islet cell aggregates are superior to islets for transplantation in microcapsules. Diabetologica.

[23] Omer AO (2004). Exercise induces hypoglycemia in rats with islet transplantation. Diabetes.

[24] Papas KK (2015). Islet oxygen consumption rate (OCR) dose predicts insulin independence in clinical islet autotransplantation. PLoS One.

[25] Papas KK (2010). Prediction of marginal mass required for successful islet transplantation. J. Invest. Surgery.

[26] Colton, C. K. *et al*. Characterization of Islet Preparations. In *Cellular Transplantation from Laboratory to Clinic* (eds Halberstadt, C. & Emerich, D. F.) 85–134 (Elsevier, Inc., 2007).

[27] Giwa S (2017). The promise of organ and tissue preservation to transform medicine. Nat Biotech..

[28] Emamaullee JA, Shapiro AM (2007). Factors influencing the loss of beta-cell mass in islet transplantation. Cell Transplant..

[29] Davalli AM (1995). A selective decrease in the beta cell mass of human islets transplanted into diabetic nude mice. Transplantation.

[30] Ryan EA (2002). Successful islet transplantation: continued insulin reserve provides long-term glycemic control. Diabetes.

[31] Rickels MR (2005). β-cell function following human islet transplantation for type 1 diabetes. Diabetes.

[32] Clatworthy MR (2013). B cell responses to allograft-more common than we thought. Am. J. Transplant.

[33] Arribas J, Borroto A (2002). Protein ectodomain shedding. Chem. Rev..

[34] Dello Sbarba P, Rovida E (2002). Transmodulation of cell surface regulatory molecules via ectodomain shedding. Biol. Chem..

[35] Albert ML, Sauter B, Bhardwaj N (1998). Dendritic cells acquire antigen from apoptotic cells and induce class I-restricted CTLs. Nature.

[36] Gallucci S, Matzinger P (2001). Danger signals: SOS to the immune system. Curr. Opin. Immunol..

[37] Shi Y, Evans JE, Rock KL (2003). Molecular identification of a danger signal that alerts the immune system to dying cells. Nature.

[38] Papas KK (2007). A stirred microchamber for oxygen consumption rate measurements with pancreatic islets. Biotechnol. Bioeng..

[39] Johnson AS (2011). Quantitative assessment of islets of Langerhans encapsulated in alginate. Tissue Engineering Part C Methods.

[40] Wilson DF (1988). The oxygen dependence of mitochondrial oxidative phosphorylation measured by a new optical method for measuring oxygen concentration. J. Biol. Chem..

[41] Avgoustiniatos, E. S. & Colton, C. K. Design considerations in Immunoisolation. In *Principles of Tissue Engineering* (eds Lanza, R., Langer, R. & Chick, W.) 336–346 (R. G. Landes Company, 1997).

